# Revisiting the neuroinflammation hypothesis in Alzheimer’s disease: a focus on the druggability of current targets

**DOI:** 10.3389/fphar.2023.1161850

**Published:** 2023-06-09

**Authors:** Maylin Wong-Guerra, Camila Calfio, Ricardo B. Maccioni, Leonel E. Rojo

**Affiliations:** ^1^ Departamento de Biología, Facultad de Química y Biología, Universidad de Santiago de Chile, Santiago, Chile; ^2^ Centro de Biotecnología Acuícola, Facultad de Química y Biología, Universidad de Santiago de Chile (CBA-USACH), Santiago, Chile; ^3^ International Center for Biomedicine (ICC), Santiago, Chile; ^4^ Laboratory of Cellular and Molecular Neurosciences, Faculty of Sciences, University of Chile, Santiago, Chile

**Keywords:** Alzheimer’s disease, druggable targets, neuroinflammation, neuroimmunomodulation, immuno-senescence, B and T cells, brain lymphatic system

## Abstract

Alzheimer’s disease (AD) is the most common form of neurodegenerative disease and disability in the elderly; it is estimated to account for 60%–70% of all cases of dementia worldwide. The most relevant mechanistic hypothesis to explain AD symptoms is neurotoxicity induced by aggregated amyloid-β peptide (Aβ) and misfolded tau protein. These molecular entities are seemingly insufficient to explain AD as a multifactorial disease characterized by synaptic dysfunction, cognitive decline, psychotic symptoms, chronic inflammatory environment within the central nervous system (CNS), activated microglial cells, and dysfunctional gut microbiota. The discovery that AD is a neuroinflammatory disease linked to innate immunity phenomena started in the early nineties by several authors, including the ICC´s group that described, in 2004, the role IL-6 in AD-type phosphorylation of tau protein in deregulating the cdk5/p35 pathway. The “Theory of Neuroimmunomodulation”, published in 2008, proposed the onset and progression of degenerative diseases as a multi-component “damage signals” phenomena, suggesting the feasibility of “multitarget” therapies in AD. This theory explains in detail the cascade of molecular events stemming from microglial disorder through the overactivation of the Cdk5/p35 pathway. All these knowledge have led to the rational search for inflammatory druggable targets against AD. The accumulated evidence on increased levels of inflammatory markers in the cerebrospinal fluid (CSF) of AD patients, along with reports describing CNS alterations caused by senescent immune cells in neuro-degenerative diseases, set out a conceptual framework in which the neuroinflammation hypothesis is being challenged from different angles towards developing new therapies against AD. The current evidence points to controversial findings in the search for therapeutic candidates to treat neuroinflammation in AD. In this article, we discuss a neuroimmune-modulatory perspective for pharmacological exploration of molecular targets against AD, as well as potential deleterious effects of modifying neuroinflammation in the brain parenchyma. We specifically focus on the role of B and T cells, immuno-senescence, the brain lymphatic system (BLS), gut-brain axis alterations, and dysfunctional interactions between neurons, microglia and astrocytes. We also outline a rational framework for identifying “druggable” targets for multi-mechanistic small molecules with therapeutic potential against AD.

## Graphical Abstract

**Figure:**
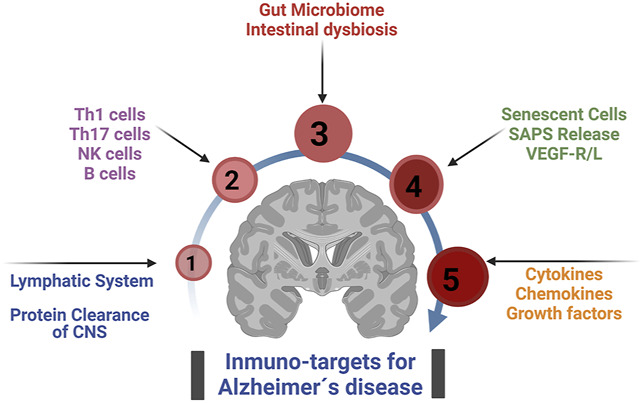
Main immuno-target for treat Alzheimer´s disease from a neuro-immunomodulatory perspective.

## Introduction

As human beings increase life expectancy in modern societies, healthcare systems face major challenges dealing with neurodegenerative disorders and achieving healthy aging (Alzheimer’s Disease International, 2020; Prince et al., 2015). Advanced medical technologies and changes in life style allowed human beings to live longer, which affects elderly today and will affect future generations. The evidence shows that the X, Z and Y generations will live much longer than the baby boomers and all generations before them (Alzheimer’s Disease International, 2020). This dramatic increase in life expectancy brings an enormous economic burden for governments across the globe (Prince et al., 2015). The global socioeconomic impact of neurodegenerative diseases, particularly Alzheimer’s disease (AD), is inevitably increasing, reaching over 100 million people by 2050 (Alzheimer’s Disease International, 2020; Prince et al., 2015; Dubois et al., 2016). There were over 55 million people worldwide living with dementia in 2020, and it is estimated that around 1 billion people are aged 60 years or older, of which over 5% suffer of some form of dementia (Alzheimer’s Disease International, 2020; World-Health-Organization, 2021). These numbers will almost double every 20 years, reaching 78 million with AD in 2030 and 139 million in 2050 (Alzheimer’s Disease International, 2020).

Although several factors such as genetic, environmental and endogenous components are known to influence AD onset and progression (Prince et al., 2015; Dorszewska et al., 2016; Onyango et al., 2016), the pathological mechanisms underlying AD are not yet fully identified. This lack of information has delayed the development and approval of effective disease course-modifying therapies. To date, few FDA-approved drugs are available for AD patients, three of which aim to merely ameliorate cognitive symptoms by inhibiting the degradation of acetylcholine (donepezil, rivastigmine, galantamine). Another drug is memantine, which inhibits excitotoxicity mediated by NMDA receptor hyperactivation (Association, 2020; Vaz and Silvestre, 2020). Severe AD cases are treated with drug combinations such as donepezil + memantine, but all these pharmacological therapies can temporarily improve cognitive functions. More recently, anti-Aβ monoclonal antibodies against Aβ aggregates, aducanumab (Aduhel^®^) and lecanemab (Leqemb^®^), were approved by the FDA (Dunn et al., 2021; Larkin, 2023). However, these immunotherapies are in the beginning of a controversial path to demonstrate safety and efficacy in AD patients.

Two central theories have marked the study of the onset and progression of AD. The well-known amyloid cascade hypothesis states that elevated levels of Aβ oligomers, polymers or insoluble amyloid plaques lead to subsequent pathological neuronal death in AD (Hardy and Allsop, 1991; Hardy and Higgins, 1992; Thinakaran and Koo, 2008). However, numerous clinical trials testing the efficacy of Aβ-based therapies in AD or mild cognitive impairment (MCI) patients have failed in improving cognitive function and memory impairment. Perhaps the monoclonal antibodies recently approved by the US FDA, aducanumab (Dunn et al., 2021) and lecanemab (Larkin, 2023), will demonstrate that anti-amyloid-based therapies can make a real impact in clinical practice. However, their efficacy and safety still remain to be established at large scale (Mahase, 2003; Association, 2020; Jeremic et al., 2021). The other central AD hypothesis focuses on the neurofibrillary pathology, initiated by the pathological misfolding and aggregation of tau protein (Braak and Braak, 1991; Fernández et al., 2008; Maccioni et al., 2010), resulting in synaptic loss, axonal retraction, and neuronal death (Wang and Mandelkow, 2016). Tau protein has physiological roles as a microtubule-stabilizing protein in axons and a synaptic protein involved in dendritic elongation, synaptic plasticity, and memory processes (Robbins et al., 2021). Therefore, significant efforts have been devoted to preventing tau protein aggregation or promoting the clearance of tau tangles. A clear connection between tau aggregates and neuroinflammation was reported by Morales et al. (Morales et al., 2013), as they demonstrated that tau oligomers and fibrils induce microglial activation with elevated production of nitrites and IL-6 (Morales et al., 2013). Similarly, amyloid aggregates are also known to induce microglial activation. One of the most accepted hypotheses regarding neuroinflammation in AD states that microglial activation is a biphasic process, which can be protective at early asymptomatic AD stages, but deleterious during the symptomatic late phases of the disease (Perea et al., 2020; Leng and Edison, 2021). Thus, the clinical progression of AD is paralleled by the transition from a protective anti-inflammatory phenotype into a disease-promoting phenotype of microglial cells (Leng and Edison, 2021). This raises the question as to how neuroinflammation can be modified to ameliorate cognitive decline in AD patients. In this review, we focus our discussion on “druggable” targets that may offer the possibility of preventing the transition of microglia into a pro-inflammatory deleterious phenotype.

Over the past 20 years, evidence from observational and epidemiologic studies has suggested that anti-inflammatory drugs would delay or even revert AD cognitive decline (McGeer et al., 1990; Martyn, 2003). Non-steroidal anti-inflammatory drugs (NSAIDs) are the main family of compounds mentioned in the literature, while corticosteroids failed to prove efficacy against AD symptoms (Wang et al., 2015). Although some studies suggested promising results, the current data is inconclusive and sometimes contradictory (Wang et al., 2015; Jordan et al., 2020; Mikhailichenko et al., 2021). Several meta-analyses agree that the quality of the observational, epidemiological, and clinical studies is a major limitation to demonstrate preventive effects of NSAIDs in AD. Long-term exposure (over 24 months) is necessary for an NSAID to show (any) protective effect, which raises the safety issue of chronic NSAIDs, especially for the non-selective COX1/COX2 inhibitors. Future clinical studies should not ignore this potential problem. In addition, the conclusions from NSAIDs epidemiological studies are limited by the differences in study design (indication, dosage, drug, pharmaceutical form). Therefore, well-designed studies and innovative approaches are still lacking to demonstrate the efficacy of NSAIDs against AD.

Basic and clinical evidence suggests that neither the amyloid cascade hypothesis, nor the aggregated tau hypothesis can solely explain the pathogenesis of AD, suggesting the involvement of other pathological processes (Prince et al., 2015; Dorszewska et al., 2016; Onyango et al., 2016). In particular, after discovering elevated levels of inflammatory cytokines, complement, and microglial activation in AD animal models (Fillit et al., 1991; Aisen and Davis, 1994; Eikelenboom et al., 1994) and AD risk genes associated with immune responses (López González et al., 2016). Thus, not only inflammation, but also, the brain immunomodulation have emerged as central players in AD pathology, making sense of the neuro-immunomodulatory hypothesis proposed by our group and others (Fernández et al., 2008; Rojo et al., 2008; Maccioni et al., 2009; Heneka et al., 2015).

In this review, we revisit and discuss the pathological hallmarks of AD analyzed from a neuro-immunomodulatory perspective, focusing on the connections between immuno-molecular targets and the classical tau/amyloid hypnotizes. We also provide a rational framework for the potential pharmacological exploitation of these targets against AD. We also discuss potential positive and negative interactions of the immune system with other biological functions to point out a rational search for modulators of neuroinflammation. We specifically focus on connecting AD pathology with the activity of immuno-senescent cells, the interactions between neurons and immune cells during AD progression, contributions of the brain lymphatic system, and the links between the gut-brain axis and neuroinflammation. Finally, we revisit the multi-target strategy and its potential contributions to new AD therapies.

### Neuroinflammation and AD: a focus on neurons, glial cells and misfolded proteins

A healthy interaction of neurons with glial cells, soluble mediators and the immune system is essential to functional properties in the normal brain, such as cognition and behavior. The Central Nervous System (CNS) has traditionally been regarded as a site of immune privilege, protected by specialized barriers. However, it is neither immunologically separated from the peripheral immune system (Sandrone et al., 2019) nor inert to damage signals from the peripheral organs. Neuroinflammation generally refers to an inflammatory response within the CNS that can be caused by various inner pathological insults (ischaemia, cellular signals, molecules) or outer factors (infections, trauma, toxins) (Fernández et al., 2008). Neuroinflammation is characterized by the activation of innate immune cells, microglia and astrocytes, permeable endothelial cells and infiltrating blood cells derived from biochemical or mechanical damage to the brain structures or the blood–brain barrier (BBB) (DiSabato et al., 2016; Gilhus and Deuschl, 2019). Thus, neuroinflammation triggers the production and release of inflammatory mediators including cytokines (IL-1β, IL-6, IL-18) and tumor necrosis factor (TNF), chemokines (CCL1, CCL5, CXCL1), small-molecule messengers (prostaglandins and nitric oxide), and reactive oxygen by the different cells of the immune system (DiSabato et al., 2016).

Some aspects of inflammation in connection with AD pathophysiology were hypothesized almost 30 years ago (Eikelenboom et al., 1994) (Rogers et al., 1996). In 1994 it was published that chronic inflammation in rats reproduced components of the neurobiology of AD (Hauss-Wegrzyniak et al., 1998) and demonstrated later by studies on the complete cascade of reactions derived from microglial activation by damage signals (Fernández et al., 2008; Rojo et al., 2008). Different studies demonstrate that Alzheimer´s disease as an inflammatory disease starts decades before the appearance of severe cognitive decay (Di Benedetto et al., 2017). Microglial activation occurs years before AD onset (Eikelenboom et al., 1994; DiSabato et al., 2016).

Studies demonstrated that a neuro-inflammatory status begins decades before the full-blown cognitive impairment of AD patients (Robbins et al., 2021). Furthermore, there is a strong link between neuroinflammation, amyloid and tau accumulation in the human brain (McGeer et al., 1990; Martyn, 2003; Wang et al., 2015; Leng and Edison, 2021). The accumulated evidence raised the question as to how neuro-inflammation can provide new pharmacological targets against AD (Fernández et al., 2008; Rojo et al., 2008; Maccioni et al., 2009).

In the context of anti-inflammatory therapies for AD, many studies attempt to show evidence of the efficacy of anti-inflammatory drugs in controlling peripheral inflammation (McGeer et al., 2018). However, none of these findings has actually influenced AD therapeutics. Some hopes are arising from the study of nutraceuticals with some anti-inflammatory activity, including quercetin (Maccioni et al., 2022) and the Andean shilajit supported by recent clinical trials (Guzman-Martinez et al., 2021). Therefore, natural multicomponent formulas are plausible, considering that they correspond to multitarget therapy for a multifactorial disease such as AD (Guzman-Martineza et al., 2001).

### Neuroinflammation and immunosenescence in Alzheimer´s disease

The concept of *Cellular Senescence* has existed for a long time, ever since A. Weismann discovered that living organisms die because tissues cannot renew forever and cell division is not ever-lasting (Zhu et al., 2015). Weismann´s findings were confirmed and further developed by Hayflick in 1961 (Hayflick and Moorhead, 1961), demonstrating that mammalian cells have a finite capacity for cell division. These non-dividing (senescent) cells display distinctive morphological and metabolic features associated with the irreversible arrest of cell proliferation and the so-called “senescence-associated secretory phenotype” (SASP) (Childs et al., 2017). The molecules secreted by cells with a SASP activate the immune system resulting in brain inflammation and the recruitment of immune cells (Lazic et al., 2022). Under normal physiological conditions, these neuro-inflammatory responses participate in numerous neuroprotective processes. However, the chronic persistence of inflammatory signals/responses in the brain leads to a pathological neuro-inflammatory environment (Fernández et al., 2008; DiSabato et al., 2016). Aging leads to numerous changes in the brain, including alterations of the immune system causing greater susceptibility to neurodegenerative diseases (Liu, 2022). Maintaining well-balanced inflammatory and anti-inflammatory homeostasis is pivotal for healthy aging. A chronic disruption of this delicate balance during aging can trigger pathological pathways in which some players of immunity deteriorate; some remain unchanged, and others become over-activated. The deleterious alterations of the immune system occurred during aging are known as immuno-senescence (Müller et al., 2019). The sustained low-grade inflammation or the so-called “inflammaging”, often observed in elderly, is a pathological phenomenon where inflammatory mediators are released by depleted or over-activated immune cells (exhausted memory T lymphocytes, monocytes, activated macrophages) that maintain a chronic inflammation status, which is associated with the onset of age-related diseases in elderly individuals, in particular AD (Bektas et al., 2017; Franceschi et al., 2017; Nikolich-Žugich, 2018).

Age is the major risk factor for AD and Parkinson’s disease (PD), the two most prevalent neurodegenerative diseases. It has already been established that the prodromal stages of AD begin decades before the cognitive symptoms are evident (Dubois et al., 2016). Although the proportion of senescent cells accumulated in the brain during healthy aging is low with respect to the rest of the brain tissue, it can reach nearly 15 percent of all nucleated cells in old primates (Zhu et al., 2015). This senescent phenotype is increased in neurodegenerative diseases compared to healthy controls. Several researchers have proposed that the clearance of senescent cells could have therapeutic use in AD (Bussian et al., 2018; Zhang et al., 2019), PD (Chinta et al., 2018) and ALS (Trias et al., 2019) models. Therefore, we propose that targeting the molecular players involved in CNS senescence can be therapeutically valuable.

**FIGURE 1:**
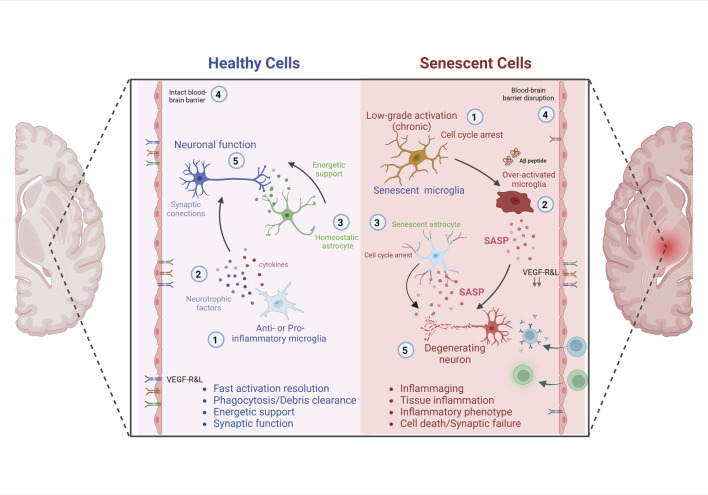
Impact of SNCs and Neuroinflammation on Neuronal degeneration and Alzheimer´s disease. Healthy and young cells, including microglia (Alzheimer’s Disease International, 2020) and astrocytes, release pro- and anti-inflammatory mediators, growth factors (Prince et al., 2015) and bioenergetic pathway mediators (Dubois et al., 2016), which contribute to the maintenance of brain homeostasis, neuronal survival and cognitive functions (Onyango et al., 2016). In conjunction with the integrity of the blood-brain barrier (World-Health-Organization, 2021), characterized by a specific receptor ratio that ensures selectivity to molecules and cells. However, senescent microglia (Alzheimer’s Disease International, 2020) and senescent astrocytes (Dubois et al., 2016) maintain a chronically higher than basal level of activation, cell cycle arrest, remain hyper activated to different stimuli with low resolution of the inflammatory response (Prince et al., 2015). These senescent cells acquire a senescence-associated secretory phenotype (SASP), characterized by the constant release of pro-inflammatory mediators, which directly affect the activation of neurodegenerative cascades, cognitive impairment and death. In addition, the senescence of elements of the blood-brain barrier (World-Health-Organization, 2021), and the decrease or alteration in the proportion of specific receptors that ensure selectivity to molecules and cells, contribute to perpetuate brain deterioration and the development of AD (Onyango et al., 2016).

Cellular senescence is characterized by an irreversible cell cycle arrest and SASP cells (Di Benedetto et al., 2017). SAPS molecular markers can be found in various brain cell types, such as neurons, astrocytes, oligodendrocytes, microglia, ependymal cells, and endothelial cells. In particular, aging microglia show a profile of an increased inflammatory status known as microglia priming, characterized by: 1) decreased activation threshold and switch to a pro-inflammatory state, 2) increased baseline expression of inflammatory markers and mediators, and 3) exaggerated inflammatory response following immune activation (Norden et al., 2015). Age-associated changes in gene expression in human microglia were studied elsewhere (Galatro et al., 2017); 212 genes were increased, and 360 genes decreased, including integrin modulators involved in cell assembly and motility (integrins TLN1 and TLN2, e-cadherin CTNNA2, profilin, F-actin). In addition, the gene expression of axonal guidance markers (DOCK1, DOCK5), cell surface receptors, microglial sensome (CXCR4, CD163, IGF2R, IFNGR1, P2RY12), growth factor VEGF-A and transcription factor RUNX3 were modified in aged human microglia (Galatro et al., 2017). These changes alter the microglia’s capacity to sense the environment and initiate chemotaxis toward injured sites, apoptotic cells, or invading microorganisms. This immune-senescent scenario seems a feasible explanation for the age-related decrease in phagocytosis and regional neuroimmune over-activation. It can explain the biphasic nature of the AD inflammation process, where early AD brain shows microglia with a healthy anti-inflammatory phenotype, whereas advanced AD stages show a toxic pro-inflammatory phenotype of microglial cells (Leng and Edison, 2021). Thus, primed microglia can exhibit an exaggerated inflammatory response to secondary sub-threshold challenges (micro-stroke, traumatic brain injury, depression, stress, metabolic dysregulation). Consequently, uncontrolled inflammatory damages can lead to cognitive deficits, impaired synaptic plasticity and accelerated neurodegeneration (Figure 1) (Norden et al., 2015).

For example, microglia responds to Aβ release from amyloid plaques through phagocytosis, TGFβ signaling, and the activation of specific receptor-ligand systems (complement receptors, Fc receptors, toll-like receptors, receptor for advanced glycosylation end products (RAGE), and triggering receptor expressed by myeloid cells 2 (TREM2)) (Agrawal and Jha, 2020). Meanwhile, tau protein also induces NOD-, LRR-, and pyrin domain-containing 3 (NLRP3) inflammasome activation, a multimeric signaling complex that activates inflammatory processes (Perea et al., 2020).

Transcriptomic analyses of aged human microglia revealed TGFβ signaling KEGG pathway was enriched for downregulated genes, this highlights the alterations of homeostatic programs as microglia trigger reactive pathways to respond to aging-related changes such as the accumulation of brain amyloid burden (Olah et al., 2018). This implies that, in humans, aged brains would be less prepared to deal with the aggregated p-tau and amyloid peptides. Of note, aged patients have shown a significantly increased expression of brain Toll-like receptors (TLRs) in the parenchymal microglia (Olah et al., 2018). AD patients and rodent AD models also exhibit similar increases in TLRs (Landreth and Reed-Geaghan, 2009). These studies suggest that increased expression of TLRs is associated with age-related inflammation. This inflammatory status and genetic AD risk factors exacerbate disease progression. In this sense, TLRs have been proposed as a promissory molecular target in aged patients with AD pathology, suppressing microglia over-activation, reducing neuronal death, and improving learning and memory function, particularly the inhibition of TLR2 (Landreth and Reed-Geaghan, 2009), TLR4 (Calvo-Rodriguez et al., 2020; Wu et al., 2022) and TLR5 (Landreth and Reed-Geaghan, 2009; Chakrabarty et al., 2018; Herrera-Rivero et al., 2019). The druggability of TLRs is still to be established, but it is worth mentioning that, in advanced AD cases, controlling TLRs expression could prevent over-inflammation. Further research on the TLR3 signaling could offer interesting possibilities for AD drug-development programs, since TLR3 is considered an essential component of various defensive responses against viral and bacterial infections and its activation induces inflammatory damage to the CNS (Li et al., 2021).

Of note, regarding the immuno-senescent scenario, age-related changes in brain endothelial cells are also connected with degenerative diseases, such as AD. Vascular Endothelial Growth Factor-A (VEGF-A) drives angiogenesis, which typically addresses blood flow reductions and global hypoxia, but also works as an innate immunity modulator. Aging decreases capillary density, flow-mediated vasodilation, and endothelial nitric oxide synthase (eNOS) function. Aging also impairs HIF1α activation, VEGF production, and inhibits matrix metalloproteinase activity (Leng and Edison, 2021). In addition, reactive oxygen species (ROS) in aged endothelial cells impair eNOS activation, decreasing the bioavailability of nitric oxide (NO) due to the uncoupling of eNOS function and increase destruction of NO. Hence, senescent endothelial cells have lower proliferative activity and cell migration capacity than non-senescent cells. This leads to reduced tube formation and vascularization, and organ dysfunction. VEGF-A (VEGF) is upregulated in response to hypoxia, and vascular dementia, and AD (Cho et al., 2017; Harris et al., 2018). The tyrosine kinase receptors VEGFR1 and VEGFR2 (vascular endothelial growth factor receptors 1 and 2) and its co-receptors mediate VEGF-A effects. Some studies suggested that angiogenesis and levels of VEGF and VEGFRs are directly linked to chronic inflammation, probably favoring cellular infiltration and proliferation (Winkler, 2000; Kirk and Karlik, 2003). Also, recent evidence suggests that aberrant VEGF-A signalling in Alzheimer’s disease may affect physiological angiogenic functions, leading to cerebral capillary stasis and reductions in blood flow as a presumed mechanism of accelerated cognitive decline (Ali and Bracko, 2022). These pieces of evidence point to the hypothesis that age-related changes in the vascular system are linked to chronic neuroinflammation, progressive deleterious impact on cognitive functions and neuronal death. The druggability of these molecular pathways in AD was suggested based on the stimulation of VEGFR1 and VEGFR2 (Cho et al., 2017; Harris et al., 2018), aiming to rescue blood flow and replace cells and molecules throughout the entire brain.

Re-activation of microglial proliferative programs is among the earliest responses to pre-pathological events in the brain, such as the accumulation of Aβ and tau aggregates in AD. This program places a subpopulation of cells called disease-associated microglia (DAM) on a trajectory toward cellular senescence (Hu et al., 2021). The inhibition of early microglial proliferation could prevent the onset of microglial senescence, which ultimately accelerates neurodegeneration associated with amyloid and tau pathology. In this context, inhibitors of the colony-stimulating factor 1 (CSF1) receptor (CSF1R) have emerged as promising drug candidates against AD neuroinflammation (Sosna et al., 2018; Hu et al., 2021). Preclinical efficacy studies demonstrated that early treatment with GW02580 (a CSF1R antagonist) inhibits microglial overgrowth, prevents microglial senescence, and reduces axonal pathology associated with the Aβ plaques (Hu et al., 2021). Another study in the 5xFAD AD murine model, using microglia-depletion strategies with long-term high doses of a CSF1R inhibitor (PLX3397), showed that microglial depletion prior to plaque formation is sufficient to prevent the onset of plaque pathology. These studies demonstrated that early inhibition of the CSF-1 receptors and microglia ablation prevent the formation of new amyloid plaques, without protecting neurons from old plaques (Sosna et al., 2018). Meanwhile, late-stage inhibition of the CSF1Rs with GW2580 is beneficial for synaptic preservation, and cognition (Dagher et al., 2015), and it turns the microglial inflammatory profile into an anti-inflammatory phenotype (Olmos-Alonso et al., 2016). The inhibition of CSF1Rs with PLX3397 also rescues tau pathology and brain atrophy in a model with combined tau and amyloid pathologies, showing that DAMs in the proximity of amyloid plaques are resistant to CSF1R inhibition.

In contrast, non-plaque DAMs and tau pathology are sensitive to CSF1R inhibition in AD murine models (Lodder et al., 2021). These results show that the CSF1Rs is promising druggable target against tau and amyloid-induced brain damage. However, it is still necessary to establish the safety profile of CSF1R antagonists since the CSF1R inhibitors lack specificity for specific cell subpopulations (Pharmaceutica, 2019; Lei et al., 2020). However, in 2019 a phase 1 clinical trial was launched to evaluate the safety and efficacy of a CSFR1 inhibitor (JNJ-40346527) in AD and mild cognitive impairment patients (Mancuso et al., 2019; Pharmaceutica, 2019). The main aims of the study are: 1) to evaluate changes in CSF levels of proteins which interact with CSF1R, 2) to measure changes in the activity or levels of affected brain microglial cells and 3) to identify biomarkers to track AD progression (Pharmaceutica, 2019).

Although astrocytes are not histologically and physiologically considered immune cells, their role in the inflammatory response is well documented (Lazic et al., 2022). Activated astrocytes can prevent neurodegeneration by forming glial scars. However, chronic activation can promote neuroinflammatory events and ultimately lead to neurodegeneration. Glial cells are involved in maintaining the brain homeostasis and neuronal synapse. Therefore, it is reasonable to expect that the astrocyte senescence is linked to age‐related neurodegenerative disease (Verkhratsky and Nedergaard, 2018; Cohen and Torres, 2019; Lazic et al., 2022). Gene expression studies suggest that aging alters regional gene expression patterns, with much greater impact on astrocytes and oligodendrocytes than on neurons themselves (Soreq et al., 2017). One of the most intriguing findings is that in the *substantia nigra* and thalamus of young subjects there is a high density of astrocyte-specific genes, which decrease significantly with aging, whereas astrocyte-specific genes with lower expression in young subjects increase significantly in various areas of the aged brain, except in the *substantia nigra* and thalamus (Soreq et al., 2017). This reflects remarkable shifts in the regional patterns of astrocyte-specific gene expression, and one of the most pronounced changes are in the movement of regional clusters from the hippocampus toward the white matter and putamen (Soreq et al., 2017). Apparently, these changes determine a less efficient and dysfunctional phenotype of astrocytes. Senescent astrocytes display permanent cell cycle arrest, altered morphology, reduced expression of nuclear lamina protein Lamin-B1, intermediate filament reorganization, increased GFAP similar to reactive astrocytes, chromatin alterations and formation of senescence-associated heterochromatic foci, up-expression and release of high-mobility group B (HMGB) proteins (Sofiadis et al., 2021), downregulation of neurotrophic growth factors, upregulation of SASP factors, and senescence-associated beta-galactosidase enzyme (SA-β-Gal) biomarker (Han et al., 2020). All factors contribute to protein accumulation, glutamate excitotoxicity, impaired synaptic plasticity, neural stem cell loss and blood–brain barrier (BBB) dysfunction (Han et al., 2020). Different studies confirmed that senescent astrocytes turn into an inflammatory phenotype (SASP-like changes) (Crowe et al., 2016; Verkhratsky et al., 2017; Han et al., 2020). Genes associated with pro-inflammatory cytokines, such as IL-6, IL-8, chemokines and proteinases are upregulated, while MHC Class II presentation related genes are downregulated in senescent astrocytes. The p38/MAPK and I-kappaB kinase/NF-kappaB cascade pathways are seemingly involved in these changes (Crowe et al., 2016). Because p38 MAPK activation is a key pathway driving senescence, (Olmos-Alonso et al., 2016), this suggests a possible convergence between the senescence program (Walsh et al., 2015) and dysregulation of immune function during aging. In addition, senescent astrocytes undergo permanent cell cycle arrest, in a process known as “replicative senescence”, p53-dependent and telomere-independent. It seems two different pathways are involved in astrocyte senescence: p53/p21waf1/CDK2 for the initiation of senescence, while the pathway involving p16/pRb family is associated with the maintenance of cellular senescence (Mijit et al., 2020). In addition, the expression of EAAT1/EAAT2 and Kir4.1 protein (involved in glutamate and potassium transport) (Limbad et al., 2020), and the water transporter AQP4 are downregulated in senescent astrocytes, leading to synaptic failure and neurodegeneration. Then, senescent astrocytes are characterized by both, lost capacity for secreting factors for the proper function of neurons and increased expression of neurotoxic of SASP factors. Senescent astrocytes accumulate in brain during aging and contribute to AD progression (Lazic et al., 2022). Yes‐associated Protein (YAP) and the cyclin‐dependent kinase 6 (CDK6) pathway are downregulated and inactivated in hippocampal astrocytes of aged and AD mice. Whereas, the activation of YAP signaling by an inhibitor of Hippo kinase MST1/2 (XMU‐MP‐1) partially rescued the senescence of astrocytes and improved the cognitive function. This provides further insights and new targets for delaying brain aging with the aim to treat aging‐related neurodegenerative diseases such as AD. These study’s results suggest that drugs that target senescent cells in the brain, might be effective treatment options for AD (Figure 1).

### Targeting B and T cells

**FIGURE 2:**
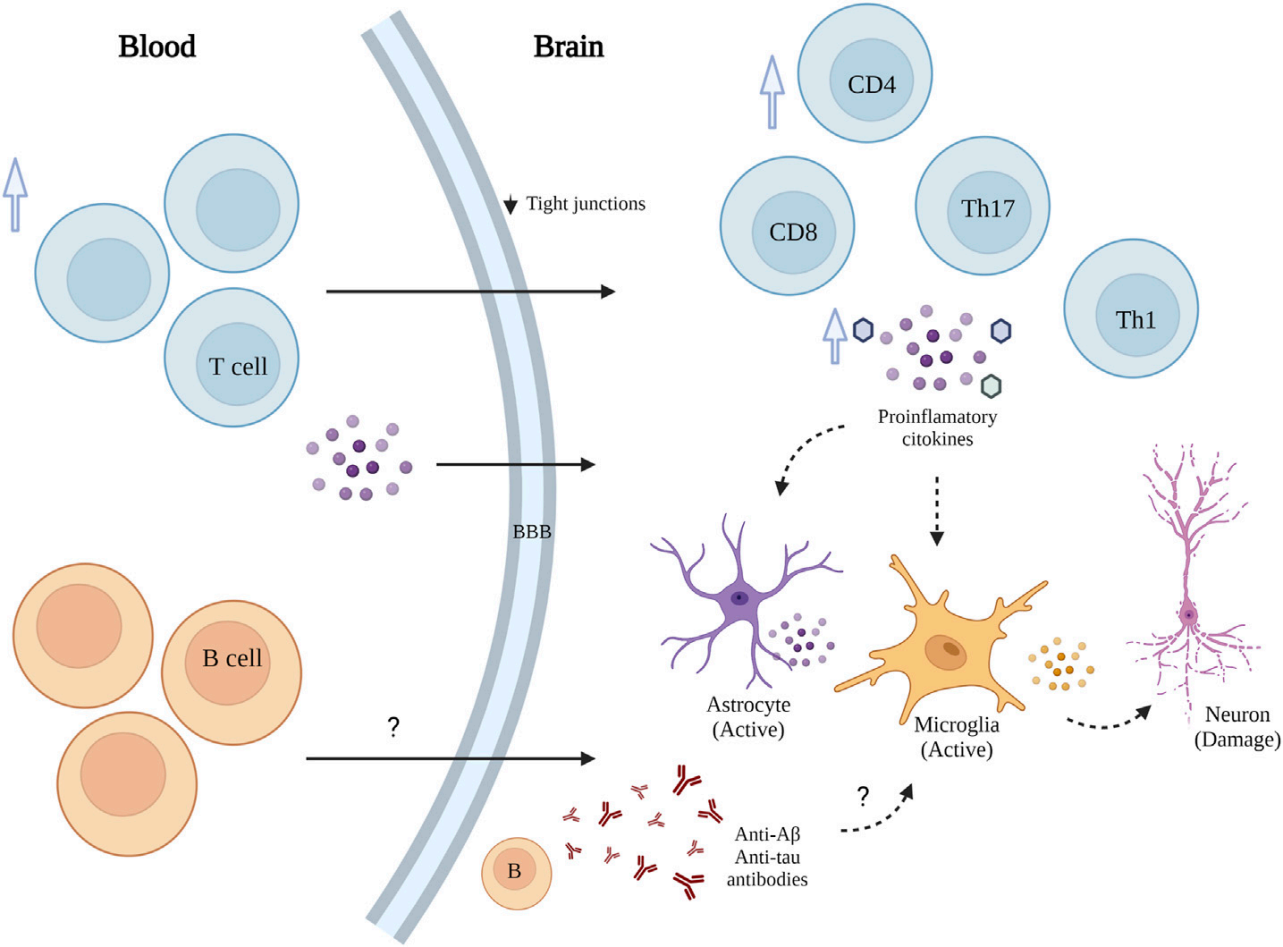
Schematic representation of T and B cells' role in AD. T and B cell infiltration into the brain exacerbates neuroinflammation and accelerates neuronal death.

Non-neuronal responses in AD have received increasing attention as relevant contributors to disease pathogenesis and progression (Lee et al., 2021). In addition to the microglia activation, the role of infiltrating peripheral immune cells (CD4^+^ and CD8^+^ T cells) in AD-associated neuroinflammation is increasingly understood (Wang et al., 2019; Dai and Shen, 2021). Peripheral infiltrated lymphocytes had been observed in the brain of both transgenic mouse models and AD patients (Bryson and Lynch, 2016). Although the role of infiltrated immune cells in AD is not fully understood (Wang et al., 2019), T cells can regulate the inflammatory response in AD through two paths that promote their penetration into brain parenchyma and exacerbate neuroinflammation (Figure 2). First, during AD, the permeability of the blood–brain barrier (BBB) gradually increases due to decreased expression of the tight junction molecules ZO1 and occluding in vascular endothelial cells (Cheng et al., 2014; Dai and Shen, 2021). Also, there is an elevated peripheral T cell expression of chemokine receptors, such as C-C motif chemokine receptor type2 (CCR2), C-C motif chemokine receptor type 5 (CCR5), and C-X-C motif chemokine receptor 2 (CXCR2) (Goldeck et al., 2013; Dai and Shen, 2021). Second, modulation of glial neuroinflammation mediated by the release of proinflammatory factors such as IFN-γ, and increase of IL-6, TNFα and IL-1 from peripheral blood mononuclear cells (PBMCs) (Mietelska-Porowska and Wojda, 2017; Dai and Shen, 2021).

Moreover, elevated peripheral type 1 and type 17-helper (Th1, Th17) cells have been associated with releasing of proinflammatory cytokines in AD (Bryson and Lynch, 2016; Cao and Zheng, 2018; Dai and Shen, 2021). Th1 cells are significant sources of INFγ, which can activate microglia and astrocytes. Th17 may induce neuronal apoptosis by releasing proinflammatory factors, such as FasL, IL-17, and IL-22 (94). In addition, CD3^+^ T cells in the brain of AD patients positively correlate with p-Tau (Laurent et al., 2018; Merlini et al., 2018).

Although the relevance of T cells in AD pathogenesis is still a matter of debate, there is convincing evidence that aberrant activation of T cells contributes to AD progression and critical AD-factors can mediate biological processes involving T cells (Zhang et al., 2013; Dai and Shen, 2021). AD risk factors, specifically Apolipoprotein E (ApoE), Aβ, α-secretase, β-secretase, γ-secretase, Tau, and neuroinflammation modulate T cell activation (Dai and Shen, 2021). Further research on these associations is critical to provide a comprehensive view of its therapeutic potential in AD. ApoE might affect T cell activation via regulation of lipid metabolism, Aβ regulates T cell function by antigen presentation, direct modulation of endogenous or exogenous Aβ, and indirect activation via monocytes. Tau also contributes to T cells activation, but the mechanism remains unclear. Secretase enzymes (α, β, γ) modulate T cell through the cleavage of specific substrates (Dai and Shen, 2021). Moreover, different T cells subsets display neuroprotective properties by regulating trophic/cytotoxic glial balance and restoring glial activation (Beers et al., 2008; Dai and Shen, 2021). CD4^+^ T cell-derived IL-10 and IL-4 are two immuno-regulatory factors with neuroprotective properties involving the inhibition of microglia with the subsequent reduction of nitric oxide and TNF-α levels (Frenkel et al., 2005). CD8^+^ T cells recruited from the periphery together with CD8^+^ Trm cells contribute to AD progression by acting on neurons/neurites directly and indirectly by affecting the functional properties of microglia (Stojić-Vukanić et al., 2020).

Another important player is the gut microbiota and its impact on brain-infiltrated immune cells. Recent evidence by Wang et al. *2019,* revealed that dysbiosis of the gut microbiota is required for the infiltration of various peripheral immune cells, including CD4^+^ and CD8^+^ T cells, B cells, natural killer (NK) cells, neutrophils, dendritic cells (DCs) and monocytes, into the brain. Among them, Th1 cells are particularly important because of their close association with the M1 microglia activation during AD progression. During AD progression, the alteration of gut microbiota composition also leads to the peripheral accumulation of phenylalanine and isoleucine, which stimulates the differentiation and proliferation of pro-inflammatory Th1 cells (Wang et al., 2019). The brain-infiltrated peripheral Th1 immune cells are associated with the M1 microglia activation, contributing to AD-associated neuroinflammation. Likewise, the elevation of phenylalanine and isoleucine concentrations and the increase of Th1 cell frequency in the blood were also observed in two small independent cohorts of patients with AD-related mild cognitive impairment (MCI). In this setting, GV-971 (a sodium oligomannate) has been proposed as a novel therapeutic strategy for AD, as GV-971 was capable of suppressing gut dysbiosis, decreasing the concentration phenylalanine/isoleucine in blood and fecal material, reducing Th1-related neuroinflammation and reversing cognitive impairment (Wang et al., 2019).

Along with T cells, B cells also play a role in promoting AD-related neuroinflammation. While immunoglobulins that target amyloid beta (Aβ) may interfere with plaque formation and hence the progression of the disease (Bulati et al., 2015; Roda et al., 2020), B cells may contribute beyond merely producing immunoglobulins. AD is associated with the accumulation of activated B cells in peripheral circulation and with the infiltration of B cells into the brain parenchyma, resulting in immunoglobulin deposits in the proximity of Aβ plaques. Interestingly, the loss of B cells alone can reduce Aβ plaque burden and disease-associated microglia and restore TGFβ+ microglia (Kim et al., 2021). Moreover, therapeutic depletion of B cells at the onset of the disease delays AD progression in murine models, suggesting that targeting B cells may also benefit AD patients.

In summary, the evidence mentioned above highlights B and T cells as essential modulators in AD-associated neuroinflammation by activating microglia and astrocytes and releasing proinflammatory factors. These anomalies suggest that controlling pathological changes in B and T cells may offer a path for immunotherapy-based targets in AD.

### Cerebral lymphatic drainage and Alzheimer’s disease

**FIGURE 3:**
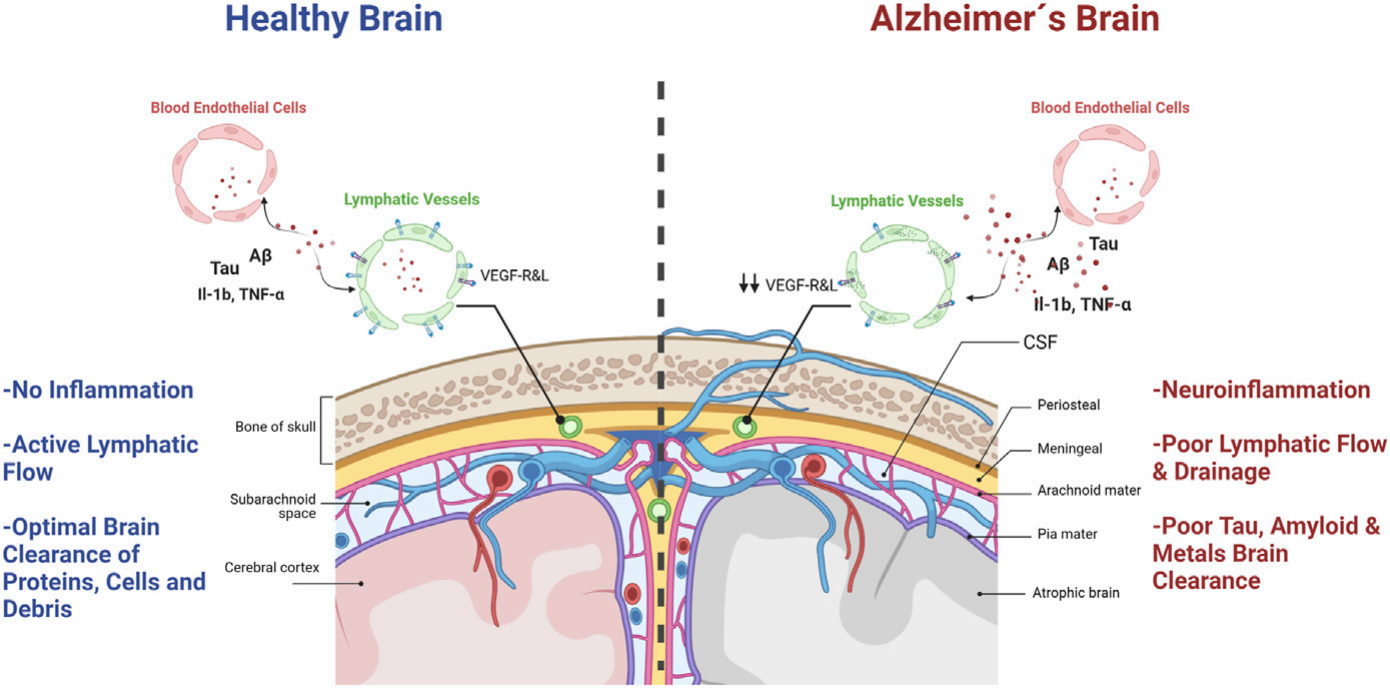
Brain lymphatic system´s role in AD. A healthy, non-inflamed brain (left panel), has a lymphatic system with an active flow that ensures optimal clearance of proteins, molecules and cells within the brain and drainage to peripheral lymph nodes. However, in AD brains (right panel), neuroinflammation and degeneration are enhanced due to poor lymphatic system flow, senescent endothelial cells, less efficient brain clearance of toxic debris, misfolded proteins, molecules and cells, and diminished brain lymphatic drainage.

Jonathan Kipnis and coworkers reported groundbreaking evidence for neuroimmunology in 2015. They demonstrated, in mice, that the central nervous system actually has a set of vascular structures with lymphatic system-like functions (Louveau et al., 2015). According to these authors, the brain would not be an immune-privileged site, as most scientists believed it. However, Kipnis’s discovery was not entirely new to medical scientists, as Dr. Paolo Mascagni described meningeal lymphatic vessels for the first time in 1787. However, the scientific community ignored his contributions for over two hundred years. Not until Sandrone et al., translated Mascagni’s original manuscripts from Italian to English and published the History of Brain Lymphatic System in 2017 (Sandrone et al., 2019), did we realize the scientific value of Mascagni´s observations. These lymphatic vascular structures were also observed in humans using modern MRI techniques (Absinta et al., 2017). These findings opened a path to explore the pathophysiological implications of the passage of molecules and immune cells in and out of the brain parenchyma. Kipnis first described this Brain Lymphatic System (BLS) in mice as a set of vascular structures that would run through the dura mater, aligned with the central venous sinuses, in close contact with the subarachnoid space (Figure 3) (Louveau et al., 2017; Da Mesquita et al., 2018a). This tubular system, located within the meninges, would not only drain molecules and cells from the brain to peripheral lymphatic organs but also offer a way for *in situ* monitoring of immune/inflammatory processes in the brain through the lymphatic liquid coming out from the brain (Sandrone et al., 2019). Such a formidable finding also opened a new research field on the role of lymphatic brain drainage in neurological diseases and its connection with peripheral immune organs (Sandrone et al., 2019). The research in this area initially focused on the role of BLS in the pathophysiology of heavily inflammatory diseases, such as Stroke and Multiple Sclerosis (Stojić-Vukanić et al., 2020). However, it quickly expanded to AD and other neurodegenerative diseases (Da Mesquita et al., 2018a; Sandrone et al., 2019).

Experiments with transgenic murine models of AD showed that the stimulation of the BLS with VEGF-c, a growth factor that promotes repair and growth of BLS structures, would protect animals from accumulating amyloid plaques in the brain (Da Mesquita et al., 2018a). Likewise, blocking BLS would aggravate brain damage induced by amyloid peptide oligomers (Da Mesquita et al., 2021). Interestingly the ablation of BLS vessels in mice with brain amyloid deposition worsened the outcome of anti-Aβ passive immunotherapy with mAducanumab16 (mouse chimeric analog of Aducanumab3) (Da Mesquita et al., 2021). Suggesting that that antibody mediated removal of amyloid aggregates would use the BLS or impaired BLS drainage would lead to a decreased passage of monoclonal antibodies against amyloid deposits from the CSF into the brain, resulting in reduced targeting and lesser clearance of Aβ plaques (Da Mesquita et al., 2021). These findings clearly demonstrated that the BLS described by Kipnis is involved in preventing amyloid oligomers-induced damage to neurons, at least in mice with brain amyloid burden.

Interestingly, worsening BLS function was associated with deleterious effects beyond histopathological markers of AD. For example, impairing BLS also induced poorer cognitive performance of AD transgenic mice in the MWM test and in the avoidance test (Da Mesquita et al., 2018a).

Although this field of research is in its infancy, and the influence of BLS on AD pathology is still a matter of active research, it is reasonable to ask the question as to how the BLS can become a “druggable” target against AD. Moreover, when numerous authors have pointed out chronic brain inflammation as an early event in AD (Fernández et al., 2008; Rojo et al., 2008; Maccioni et al., 2009), thus; it seems reasonable to hypothesize that VEGFS-c type agonists could ameliorate AD symptoms in late disease stages, or when the amyloid and tau burden in the brain tissue is high enough to overcome the BLS drainage capacity. In terms of “druggability” of the BLS in AD, it is plausible that VEGFR 3 (Vascular endothelial growth factor receptor 3), a member of VEGFRs family involved in developing and establishing new lymphatic system structures (Secker and Harvey, 2021) may become a pharmacological target against AD. This is an interesting hypothesis, since VEGFR3 ligands have already been researched for therapeutic applications in other diseases (Leppänen et al., 2013). The current knowledge on the structure of VEGFR3 binding domains allows for the rational identification of new peptides with selective VEGFR3 binding properties.

From our perspective, the disappointing clinical results of most amyloid-based therapies make it challenging to find drugs aimed at BLS-based targets that promote brain amyloid clearance.

Another critical question that remains to be answered regarding the BLS in AD is whether the manipulation of brain lymphatic drainage could modify levels of AGES-activated microglia, phosphorylated tau protein, activated T lymphocytes, pro-inflammatory cytokines, toxic metals, and oxidized lipoproteins (ox-LDL). Selective VEGFR3 ligands have shown to improve clearance of immunogenic epitopes from brain (Leppänen et al., 2013; Cho et al., 2017), which suggest a path to an improved removal of aggregated proteins and better immuno-surveillance of AD brain tissue.

In summary, it seems possible that the positive effects of VEGF-like compounds observed in AD models are associated with an improved drainage capacity of the BLS, which only becomes clinically evident in advanced stages of AD, when tau and amyloid accumulation overcomes neuronal plasticity and BLS clearance. This is consistent with the effects observed by Kpnis in animals treated with VEGF-c, in which cerebral lymphatic drainage improves molecular and cognitive markers of AD. Altogether, the BLS is seemingly a promising target to tackle in the early and late stages of AD.

## Concluding remarks

To our interpretation, there are at least four major neuro-inflammatory approaches to modify AD progression. The first is the integrity of BLS and its influence in AD pathology (Louveau et al., 2016; Louveau et al., 2017; Da Mesquita et al., 2018a; Da Mesquita et al., 2018b). Although this approach has not yet been explored as a druggable target, the current literature shows that, in murine AD models with heavy amyloid burden, the intervention of BLS improves behavioral outcomes and decreases brain amyloid aggregates. The relationship between cerebral tau pathology and the BLS integrity is certainly an important question to be answered. It is likely that protecting the function of BLS will benefit the clearance of tau oligomers and the cerebral immune function. Especially in view if the connections between peripheral and central immunological players.

The second group of potential targets for AD comprises soluble inflammation-associated ligands (harmful interleukins, cytokines and chemokines), T and B cells within the brain parenchyma. The interaction between neurons and microglia is the center for this approach. Chronic abnormal neuron-microglia interactions can prolong long enough to induce irreversible neuron damage. Here the aggregation of tau protein and amyloid peptides seems critical for promoting chronic microglial activation.

The third component is senescent status of neurons, astrocytes and glial cells. Cellular senescence is characterized by an irreversible cell cycle arrest and a pro‐inflammatory SASP (Bektas et al., 2017; Childs et al., 2017; Di Benedetto et al., 2017; Lazic et al., 2022). Although there are several targets involved here, some of them are worth more attention as potential targets for AD therapeutics: 1) Increased expression of TLRs, diminished endothelial cells, 2) downregulation of VEGF-s, and 3) increase levels of DAMs (Da Mesquita and Kipnis, 2017; Mijit et al., 2020). In addition, the over-activation of CSF1-R is a promising target (Absinta et al., 2017; Lei et al., 2020). Indeed, the first clinical trial to evaluate a CSF1-R antagonist in AD patients is currently recruiting patients. Also, increased brain levels of IL-6, IL-8 and decreased levels of MHC-II molecules are also interesting alterations to tackle.

The forth component of this puzzle is the participation of the gut-brain axis in AD pathogenesis and progression. This area has been elusive to experimental verification due to technical problems with establishing specific microbiome species and strains in AD patients and controls. There are also confounding factors in human clinical studies (different diets, life style, use of antibiotics, etc.). However, a recent report demonstrated that intestinal microbiota-mediated and systemic immune aberrations contribute to the pathogenesis of AD in ADLPAPT (amyloid, tau and neuroinflammatory AD murine model) (Kim et al., 2020). These authors suggest that there is a strong connection between the gut, blood and brain in AD, since frequent transfer and transplantation of the fecal microbiota from wild type mice into ADLPAPT mice ameliorated the formation of amyloid β plaques, neurofibrillary tangles, glial reactivity and cognitive impairment (Kim et al., 2020). So far, there is no evidence of successful fecal transplant in AD patients. However, Ueda and coworkers found that intestine levels of *Faecalibacterium prausnitzii* (F. prausnitzii) correlates well with cognitive scores and is decreased in humans with MCI compared with a healthy group (Ueda et al., 2021). Bacterial strains isolated from these healthy subjects (live Fp360 and pasteurized Fp14) were fed orally to mice inducing a significant reduction in alternation behavior in the Y-maze test compared control counterparts (Ueda et al., 2021). This is an interesting path to walk to find new probiotics with neuroimmune protecting properties.
